# Deciphering trophic interactions in a mid-Cambrian assemblage

**DOI:** 10.1016/j.isci.2021.102271

**Published:** 2021-03-04

**Authors:** Anshuman Swain, Matthew Devereux, William F. Fagan

**Affiliations:** 1 Department of Biology, University of Maryland, College Park, MD 20742, USA; 2 Department of Earth Science, Western University, London, ON, Canada

**Keywords:** Biological Sciences, Evolutionary Biology, Paleobiology

## Abstract

Exceptionally preserved fossil sites have allowed specimen-based identification of trophic interactions to which network analyses have been applied. However, network analyses of the fossil record suffer from incomplete and indirect data, time averaging that obscures species coexistence, and biases in preservation. Here, we present a high-resolution fossil data set from Raymond Quarry member of the mid-Cambrian Burgess Shale (7,549 specimens, 61 taxa, ∼510 Mya) and formulate a measure of “preservation bias” that aids identification of assemblage subsets to which network analyses can be reliably applied. For these sections, abundance correlation network analyses predicted longitudinally consistent trophic and competitive interactions. Our analyses predicted previously postulated trophic interactions with 83.5% accuracy and demonstrated a shift from specialist interaction-dominated assemblages to ones dominated by generalist and competitive interactions. This approach provides a robust, taphonomically corrected framework to explore and predict in detail the existence and ecological character of putative interactions in fossil data sets.

## Highlights

• Network analysis of well-preserved fossil communities can predict probable interactions
• Clustering analyses of these interactions reveal possible ecological categories
• Agent-based models can help infer/map these categories to known ecological patterns
• High agreement of predictions to hypothesized trophic interactions from literature

## Graphical abstract

**Figure:**
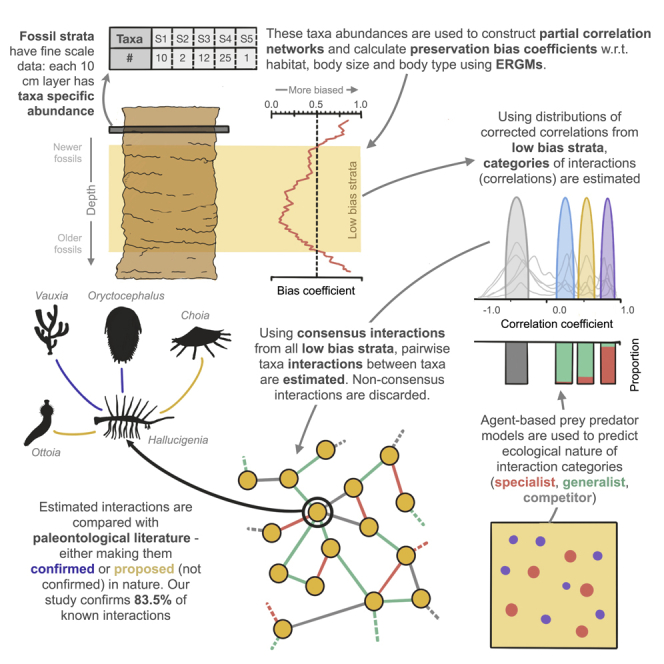

## Introduction

Evolutionarily, the Cambrian Period (541–485 Mya) is unique because it witnessed the emergence and rapid diversification of phylum-level extant animal body plans and featured the highest morphological and genetic rates of animal evolution (Erwin et al., 2011; Lee et al., 2013). Morphological disparity and behavioral complexity increased (Seilacher et al., 2005; Carbone and Narbonne, 2014), prompting hypotheses about major shifts in ecological interactions and trophic structure during this period, due to major changes such as widespread predation and active (vertical) burrowing, which may have facilitated the first complex “modern” food webs (Morris, 1986; Vannier and Chen, 2005; Dunne et al., 2008; Erwin and Valentine, 2012; Mángano and Buatois, 2014). “Conservation lagerstätten” sedimentary deposits, featuring exceptional fossil preservation of both “soft” and “hard” body features (Orr et al., 2003), permit detailed studies from which species interactions can be deduced (Butterfield, 2003). Previous works have focused on these well-preserved Cambrian localities for performing detailed paleoecological analyses and have contributed immensely to our understanding of the Cambrian ecosystem (Caron and Jackson, 2006; Caron and Jackson, 2008; Zhao et al., 2014) However, network-based approaches can provide new perspectives, helping to resolve ongoing debates and shedding new light on community structure.

Network-based studies provide critical insight on the structure and function of ecological systems (Ings et al., 2009; Poisot et al., 2016; Delmas et al., 2017), but paleo-assemblages often suffer from incomplete and indirect data (Roopnarine, 2010; Shaw et al., 2021), time-averaging across large stratigraphic sections that obscure species coexistence (Kidwell et al., 1991; Dunne et al., 2008; Roopnarine, 2010; Muscente et al., 2018), and biases in preservation, collection, and identification of both specimens and interactions (Koch, 1978; Smith, 2001; Dunne et al., 2008). Although some previous network studies have been performed on almost census preserved communities, such as in the Ediacaran (Mitchell and Butterfield, 2018; Mitchell et al., 2019; Muscente et al., 2019). Here, we report an extensive mid-Cambrian fossil abundance data set featuring excellent preservation with high stratigraphic resolution, consistent taxa presence, and low biases in collection and identification. Using partial correlation network analyses of fossil abundance data and agent-based models, we find statistical evidence recapitulating 71 of 85 previously known or suspected species interactions, propose 117 previously unknown putative interactions, and identify a shift from assemblages dominated by specialist interactions to ones dominated by competition and generalized interactions. All results derive directly from fossil abundance data, without assuming any prior knowledge about species interactions.

**Figure 1:**
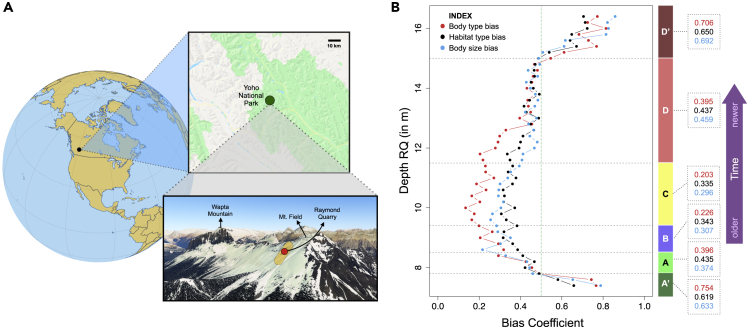
Location, biofacies, and bias (A) Location of Raymond Quarry (RQ) (Yoho National Park, BC, Canada), denoted by red dot; yellow region denotes extent of major Burgess Shale localities; samples were collected from the RQ member along the “fossil ridge” connecting Mt. Field and Wapta Mountain. (Figure S1 and methods) (B) “Preservation bias coefficient” for body type (with respect to soft, intermediate, and hard bodied categorization) (in red), habitat type (in black), and body size categories (in blue) calculated using networks in running time frame analysis, plotted along with estimated boundaries for the distinct sub-assemblages (A-D) in the 9.3 m shale section using variations of two different statistical approaches for biofacies detection: ANOSIM and SHEBI (Figure S2 and methods) and the associated average bias coefficient for each sub-assemblage (in their respective index colors for each type of bias). The green dotted line depicts the bias threshold we adopted for inclusion versus exclusion of sample layers. Preservation bias coefficients exceeding 0.5 translate into substantial changes in the structure of interaction networks calculated from abundance correlations (Figure S3B). Note that the 10 cm layers comprising regions A′ and D′ were originally identified as belonging to sub-assemblages A and D, but fossils from A′ and D′ were not used in the analyses presented here because of evidence for high levels of preservation bias among taxa.

Employing classic tools of network analysis, we characterize fine-scale structure and dynamics of the paleoecological system represented by a 7,549-specimen data set from the Raymond Quarry (RQ) of the Middle Cambrian Burgess Shale of SE BC, Canada (∼510 Mya, Figures 1A; S1). This data set, which represents one of the most complete views of early animal assemblages, consists of species-wise abundance for 61 taxa in 10 cm levels across 9.3 m of shale. These Burgess Shale assemblages are thought to be the result of rapid entombment of fossils in fine-grained sediments (Gaines et al., 2012). Oxidant flow into sediments was prevented by the quick sealing of sediments by widespread carbonate cements (a result of highly alkaline Cambrian oceans), in an ocean where low sulfate and low-oxygen bottom waters already reduced oxidant concentration. This resulted in exceptional preservation of organisms and provided a unique fingerprint of the Cambrian. Interpretation of the details of the entombment and preservation processes differ among studies (Gaines et al., 2012; Schiffbauer et al., 2014; Sperling et al., 2018).

Previous network studies of paleontological data from the Burgess Shale (e.g., Dunne et al., 2008; Shaw et al., 2021) have focused on the Walcott Quarry, which is from the same geological period and has much higher species diversity than RQ but unlike RQ lacks consistent species preservation throughout its constituent bedding planes (Devereux, 2001). In addition, all RQ bedding planes were scanned thoroughly and fossils were identified and labeled immediately upon discovery in the field to nearest decimeter bedding plane, resulting in a low collection bias (for details, see methods). Therefore, this data set's fine-scale spatial resolution and the site's exceptional, consistent preservation of soft-bodied organisms allows us to assume representative population preservation throughout (most of) the assemblage and therefore offer advantages not available to most earlier paleo-assemblage network analyses (Dunne et al., 2008; Dormann et al., 2017). Moreover, in addition to usage of network methods, we utilized agent-based models to quantify and test key concerns, affording (a) a computational approach to understand preservation bias in fossil assemblages, (b) identification of putative interactions among taxa, (c) categorization of putative interactions into ecological roles, and (d) understanding of trophodynamics over time.

## Results and discussion

Based on consensus results from two statistical approaches commonly used to define boundaries between fossil assemblages—SHEBI (Buzas and Hayek, 1998) and ANOSIM (Clarke and Warwick, 1994) (methods and Figures 1B; S2)—we identified four distinct sub-assemblages (named A-D in decreasing order of age), which match previous biofacies identification based on paleontologically defined trophic nuclei (Devereux, 2001). Based on these results, we calculated statistically corrected pairwise correlation of abundance for all taxa in each of the four sub-assemblages, and each of the 46 groupings of 20 cm levels organized to facilitate analyses at a finer stratigraphic resolution (hereafter referred to as the running time frame analysis; for the bulk of the analysis, we use these planes as constituents of the larger A-D layers/sub-assemblages, and for the “running time frame/finer stratigraphic resolution analysis”, we used data at a smaller cross section of the entire sub-assemblage, again whose smallest unit is fossil abundance in 20 cm level stratigraphic bedding planes.) (methods). These correlations, with relevant regularization, were then used to construct correlation networks, for each sub-assemblage and each component of the running time frame analysis. In these networks, each node was a taxon and each edge between a node pair represented significant correlation and thus possible interaction (methods).

Correlation networks can yield insights into possible interactions among taxa (Zhang, 2011; Barberán et al., 2012; Carr et al., 2019), but network features can be obscured by preservation and collection biases (Dunne et al., 2008; Jordano, 2016; Carr et al., 2019). Intensive sampling and detailed annotation reduced collection bias in this data set, but preservation bias can still yield altered patterns of abundance. Statistical corrections have addressed some issues of fossil preservation biases (Mitchell, 2015; Starrfelt and Liow, 2016; Flannery Sutherland et al., 2019), but these have not targeted applications involving network analyses. Moreover, previous work has shown that despite these biases, abundance and rank abundance of species in the fossil record tends to have high fidelity (see Kidwell, 2001; Kowalewski et al., 2006).

Preservation bias can occur for several reasons, most notably presence/absence of hard body parts/biomineralizable structures (which aid preservation), body size (which determines amount of preservable material and often the size of populations), and location/habitat (which provide differential conditions for preservation). If two taxa are both well preserved, their true abundance correlation is expected to exhibit less noise than correlations among pairs of taxa in which at least one taxon is not well preserved. Differential preservation biases among taxa could introduce subtle structuring in a correlation-based network that would be biased toward more well-preserved taxa. The network-level consequences of such biases can be quantified by comparison with exponential random graph models (ERGMs), which have been used to understand the effects of bias and missing data (Robins et al., 2004).

To understand the influence of preservation bias on fossil correlation networks, we constructed an agent-based simulation model (ABM) of a complex resource-prey-predator system (consisting of 17 prey, 8 predators, and a common base resource for prey; methods). We ran 1 million simulations of this ABM that differed in various initial conditions, such as starting population size of each species and average resource density. For each of the 1 million simulations, we then created 100 cases, where each of the component species was assigned independently to one of three categories differentiated by probability of preservation (methods), and also retained the corresponding base case in which each species had perfect preservation (i.e., the original abundance data). For all 101 million cases (100 million cases with modified preservation and the corresponding 1 million original cases with perfect preservation), we then calculated abundance correlations among species pairs and constructed regularized correlation networks. We formulated a bias coefficient, using ERGMs and Hamming distance, to capture the effect of differential preservation on network structure through pairwise analyses of corresponding cases with modified and perfect preservation (Figure S3, methods). Higher bias coefficients corresponded to greater alterations of network structure.

We then applied this bias coefficient to the fossil data in the running time frame analysis (methods). We separately considered three factors that could map onto differences in preservation: body type (hard bodied, partially hard bodied, soft bodied), body size (<15cm, 15–30 cm, and >30 cm maximum size), and habitat usage (endobenthic/epibenthic, nektobenthic, and nektonic/pelagic) (methods). Information on these factors was compiled from literature surveys: body type, maximum body size, and habitat usage (see Supplemental Information and, Royal Ontario Museum Database).

For all three factors, we found evidence for significant differential preservation bias at the start and the end of the collected assemblage in regions A′ and D′ that were, respectively, originally part of sub-assemblages A and D identified through biofacies detection (Figure 1B). Because of their heightened preservation bias, which was strong enough to substantially alter apparent network structure, data from regions A′ and D′ were excluded from further analyses. In contrast, we found low levels of preservation bias coefficients in each of the defined sub-assemblages (A-D), with respect to body type, body size, and habitat (Figure 1B; bias coefficient was <0.5 for all cases; methods). These preservation biases were low enough to have inconsequential effects on network structure (Figures 1B and S3). The three preservation bias coefficient series (shown in Figure 1B) are weakly correlated. This might be due to the fact that the number of specimens affects our ERGM values, and in the beginning and the end of the data set, we see low number of total specimens per level. One would expect heightened levels of preservation bias at the beginning and end of a fossil bed if the strata above and below the sampled assemblage did not allow proper preservation of organisms due to a change in environmental (preservation) conditions (Orr et al., 2003). From a taphonomic viewpoint, factors such as differential transport experienced between taxa and between fossil beds, the degree of time averaging, and pre-burial transport distances may shape the preservation of discoverable assemblage contents as well (Olson et al., 1980; Martin, 1999). Consequently, complete preservation of all ecological information is seldom expected (Flannery Sutherland et al., 2019; Saleh et al., 2020). However, the consistency in low preservation bias coefficient with respect to body preservation type, habitat type, and body size throughout sub-assemblages A-D (with removal of A′ and D′ and corresponding running time frames) suggests that the net taphonomic effect resulted in an overall relatively homogeneous burial of a group of taxa across the whole assemblage (Figure 1B). Nevertheless, some loss of taxa may not have been inferable from the fossil data using our methods, and small differences in preservation may still be present throughout the assemblage at the taxon level.

Even though we report no significant preservation biases beyond those at the A′ and D′ ends of the RQ assemblage based on the predicted “in situ” preservation potential of the RQ taxa, the original interactions may still have been subjected to certain biases (Butterfield, 2003). However, these possibilities are not pertinently different than methodological biases affecting recent or extant ecological data (Dunne et al., 2008; Armitage and Jones, 2019).

**Figure 2:**
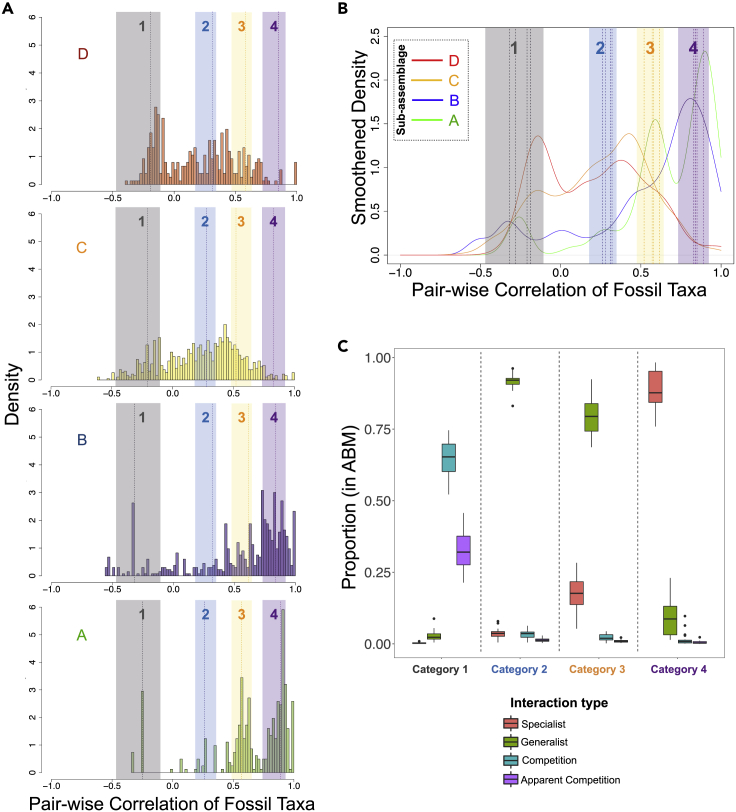
Characterization of interactions (A) Distribution of pairwise correlations across A–D; dotted lines indicate the means of the basis Gaussians in each sub-assemblage calculated using maximum likelihood analysis for decomposing the abundance correlation distributions; colored bands indicate the four interaction categories 1–4, which represent the range of the Gaussian means for each interaction type, clustered from the basis Gaussians of the running time frame analysis using spectral analysis with gap statistics (see methods). (B and C) (B) Summary of the four categories of interactions, calculated from spectral analysis, on the smoothened density distributions of pairwise correlation of fossils in the four sub-assemblages A–D; dotted lines indicate the means of Gaussian basis distributions from A–D; (C) Proportion of different feeding types in the ABM whose abundance correlations fall in the colored bands identified in (A), suggesting, for example, that the negative correlations in category 1 (gray) are dominated by competition and apparent competition interactions, whereas the strongly positive correlations in category 4 (purple) derive from specialized interactions.

Both experimental and theoretical studies predict that prey-predator abundances should be correlated on long time scales (Tobin and Bjørnstad, 2003; Liebhold et al., 2004; Blasius et al., 2020), and we found this to be true for our ABM simulations (Figure S4). This result supports the premise that fossil abundance correlations might correspond to potential species interactions, where the degree of preservation bias is low, such as in extremely well-preserved assemblages like the Burgess shale (Saleh et al., 2020) and census-like preservation of Ediacaran communities (Mitchell et al., 2019). Furthermore, the distribution of abundance correlations should characterize system-level interactions. For example, we might hypothesize that abundances of competitors should be negatively correlated, whereas abundances of species engaged in highly specialized interactions should be strongly positively correlated, assuming homogeneous transport and burial. The shape and location of the distribution of fossil abundance correlations differed among sub-assemblages A-D (Figure 2A and 2B). In particular, the frequency of small magnitude correlations and of negative correlations increased over time from A to D. Moreover, in addition to species interactions, these correlations can also be a result of habitat overlap or exclusivity (and other phenomena such as mutualisms, indirect environmental effects, etc.) even in the case of relatively homogeneous burial. Later in this work, we try to tease apart if habitat preferences had any effect on the correlation structure and found none.

To explore if changes in correlational distributions represented a shift in the dominant mode of species interaction over time, we decomposed the corrected correlation distribution for each sub-assemblage A-D, which were used in network construction, into its respective basis functions using maximum likelihood (methods). In each sub-assemblage A -D, the distribution of abundance correlations was best fit by a sum of four Gaussian distributions (Figure S6 and methods), and across sub-assemblages, the four Gaussians had similar means but different amplitudes and variances (Figure 2A). We found a similar result for the (finer stratigraphic resolution) running time frame analysis. To categorize these Gaussians in the correlations of fossil data into possible clusters, we calculated a pairwise similarity matrix of all the component Gaussians across the assemblage and then performed a spectral analysis with the “gap” statistic (Tibshirani et al., 2001; methods) on it, which revealed four clusters of Gaussians (Figures 2A and 2B; methods).

To understand the origins and potential meaning of the four clusters/categories of Gaussians in the empirical data, we used the ABM to simulate the dynamics of hypothetical ecological communities that differed in the importance of prey-predator interactions and competition. We considered trophic-relation-based ABM systems involving prey, specialist predators, and generalist predators, which implicitly also allow for competition and apparent competition (or, intra-guild competition). We compared where the abundance correlations associated with specialist, generalist, and competitive pairwise interactions from the simulated ABM communities fell relative to the four categories obtained via clustering from the fossil correlation distributions. Fully 86% of all correlations derived from interactions in the ABM fell within the intervals of the four empirically defined categories (Figures 2A–2C). In support of our initial ideas about the relationship between abundance correlation and interaction type, we found that ABM interactions involving competition and apparent competition dominate category 1, generalist prey-predator interactions dominate categories 2 and 3, and specialist prey-predator interactions dominate category 4 (Figure 2C). To explore, we used a second ABM in which each prey-predator interaction was weighted by the predator's preference for that prey. From these simulations, we recovered the specialist-generalist spectrum of interactions and further identified a non-linear relation between a predator's preferences for prey and the abundance correlations recovered from the ABM. The abundance correlation between a predator and its lower preference prey was weaker than that expected for the same prey unweighted by preference (Figure S4). In both the ABM models, the final correlations in a wide variety of parameter combinations/conditions exhibited strong effects only from the prey preference (in the second ABM) or from categorization of trophic interactions (in the first ABM where we differentiated specialists from generalists). Other initial conditions and parameters had very small influences on the correlations (see Figure S11)—rendering our results and inferences robust.

With reference to the ABM results, we can interpret that “category 1” (gray) involves negative correlations suggesting competition and apparent competition interactions among taxa (Figure 2C, leftmost column). Alternatively, such negative correlations can also arise if species have different habitat preferences and the relative availability of different habitats changes over time. Similarly, correlations in categories 2 (blue) and 3 (light orange) likely involve generalist consumers. If a consumer is not specializing on one resource but eats many, it will be only loosely correlated with its prey (Figure 2C, middle columns). A weak positive correlation could also mean that both members of a species pair use similar resources. Category 4 (purple) would derive from component Gaussians that feature consistently strong, positive correlations. This category likely represents specialist predation in which a predator depends solely or very strongly on a particular prey species (Figure 2C, rightmost column). Alternatively, if two interacting species are in a mutualistic relationship or exhibit a strong joint dependence on environmental conditions, similarly strong positive correlations could emerge. Please note that throughout our work, we will refer to interactions as specialist or generalist (or competitive) without ascribing roles to particular taxa as we cannot ascertain which species are the prey and the predator in a given pair on the basis of abundance only. Therefore, our network is undirected.

We acknowledge that correlations may exist based on similar habitat/environment and species interactions such as mutualisms that are, unlike the focus of our efforts, neither trophic nor competitive in nature. Indeed, disentangling trophic from non-trophic interactions is overall a more difficult task and would require further paleontological interaction data as well as simulation and analysis work that are beyond the scope of the current analysis.

To look at the effect of habitat, we first calculated the preservation bias for habitat (Figure 1B) and found no significant effect of habitat on the network structure. Next, to support our trophic ABM analysis as a benchmark for categorization, we tested whether two alternative reasons for correlations (i.e., habitat specializations for negative correlations and habitat/environment sharing for positive correlations) impacted our analysis of the fossil data. To do this, we used a stochastic block model (SBM) on the sub-assemblages and on the running time frame data to see if the clustered distributions of interactions could be explained instead by habitat/environment and motility data hypothesized in literature (see Figure S10).

SBMs find community structure in networks (i.e., blocks of nodes which interact more among themselves than with others outside the block) and in this application would indicate clustering of interactions based on similar habitat or motility (Karrer and Newman, 2011). To implement the SBMs, we first computed the Shannon's equitability index of each block in a given network and then calculated the weighted average of this index across blocks. An index value of 1 implies equal distribution of habitat or motility types across blocks, while a 0 indicates complete dominance of one type in a block. The SBM analyses revealed no strong dependence of the empirical correlations on either habitat or motility (Figure S10C).

Lastly, to explore whether negative correlations can arise from species specializing on different habitats, we compared the frequency of negative correlations within the same habitat to the corresponding values for different habitats. For the running timescale data, there were no more negative interactions among taxa from different habitats than from the same habitats (Pearson's *Χ* ^2^ with Yates' continuity correction: mean p value [across the entire running time frame analysis] = 0.89, range = [0.68,0.93]), thereby excluding any strong effect of habitat exclusivity. We found similar results for positive correlations, again finding an absence of association between interactions and species' habitat types (Pearson's *Χ* ^2^ with Yates' continuity correction: mean p value [for all time frame analysis] = 0.26, range = [0.19,0.35]). Collectively, these results suggest that habitat did not play a significant role in the structure, value, and distribution of correlations in our network and that these correlations instead likely stem from species interactions. Note that in our case, we are assuming that in absence of discernable effects from habitat bias, body type bias, or size bias, the statistically corrected, inferred interactions are either trophic or competitive in nature only. Future data and work are necessary to tease apart other forms of species interactions.

Refocusing on the distributions of correlations, we observe that across fossil sub-assemblages A-D, negative interactions increase in relative frequency over time (Figures 2A and 2B). Similarly, specialist and generalist interactions increased from sub-assemblage A to B, but specialist interactions are largely absent in D and occur only infrequently in C. Systematic change in the fossil transport regime could explain this, but this seems unlikely given the consistency of pairwise correlation signals over time (see Figure S9). Alternatively, long-term environmental change could have decreased regional productivity and made resources rare in the area of fossilization. Such long-term loss of productivity could have led to an increase in competitive interactions and loss of specialist interactions (including both mutualists and trophic interactions), which are more prone to extinctions (or extirpations) (Ryall and Fahrig, 2006; Colles et al., 2009). These results contrast with previous food-web-based studies, where generalists dominate early structuring of food webs, followed by specialists (Piechnik et al., 2008). As such, instead of representing colonization of new habitats, our data set may provide a window into fluctuating ecological abundances transported locally in a near-shore habitat, which were fossilized during intermittent episodes of exceptional preservation. Although we do not know the time frame of deposition of RQ exactly—and it might be fairly long—there are no anatomical changes observable in the fossil taxa, which are consistently present throughout the assemblage. This suggests that the assemblage remains within an ecological regime rather than reflecting evolutionary time.

**Figure 3:**
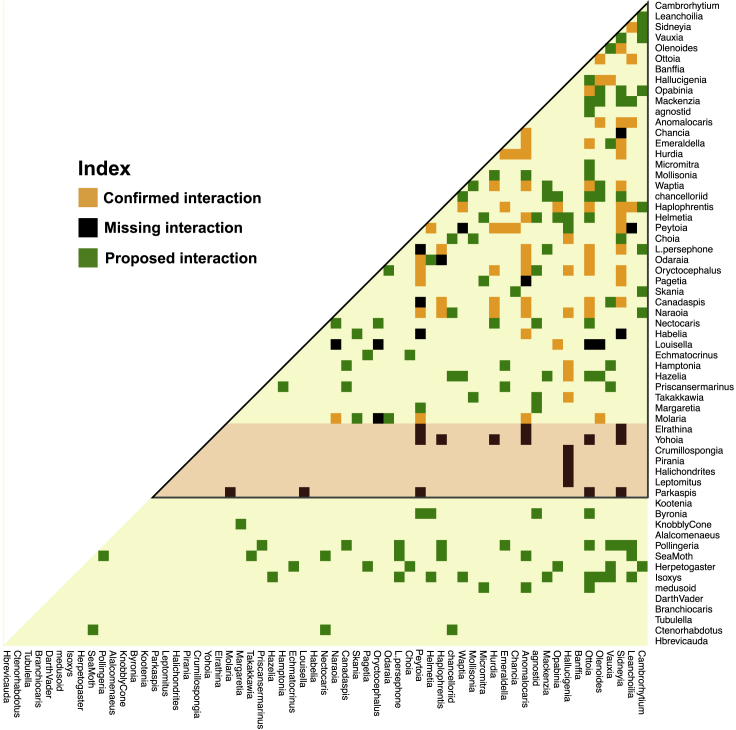
Inferred species interactions Species interaction half-matrix showing consensus interactions from our analysis, as compared with known trophic interactions from literature (Butterfield., 2003; Dunne et al., 2008; Erwin and Valentine, 2012). Confirmed interactions were proposed in the literature and supported in the correlation analyses here. Missing interactions are reported elsewhere but did not obtain any support from our abundance correlation analyses. Proposed interactions are not currently known from the paleontological literature but are suggested by analyses here. The subset of species interactions inside the black triangle were posited in previous studies (Dunne et al., 2008). The species within the light orange area were numerically rare in our data set and no statistically robust prediction could be made regarding their interactions. For classification of confirmed interactions, see Figure S8, and for consistency of interaction, see Figure S9.

Analyses of abundance correlation networks recovered many proposed prey-predator interactions. We used the term “consensus interactions” to refer to those species pairs whose abundance correlation yielded the same interaction categorization for a majority of the strata (>50%) where the two taxa co-occurred (see Figure S9 for details). For species whose trophic interactions have been described or suggested in past literature and whose abundance in this data set was sufficient for correlation analysis (Figure 3, innermost region), fully 83.5% (71 of 85) of consensus interactions predicted through our correlational analyses have been independently proposed in paleontological literature (collected from Dunne et al., 2008; Erwin and Valentine, 2012; see methods). Our analyses supplement these expert propositions by assigning pairwise interactions into categories of prey specialization or preference by reference to correlation categories (Figure 2). Furthermore, we propose 117 new possible pairwise trophic interactions based on abundance correlations identified here. These include 75 putative interactions for species whose trophic interactions were previously only partially known (Figure 3, innermost submatrix) and another 42 putative interactions involving species whose trophic interactions were not previously reported. Lastly, 18 pairwise interactions previously known from the literature could not be recovered here because the taxa involved were represented at very low densities in the fossil data set (Figure 3, submatrix with red background).

To put these results into better context, we can take the example of the enigmatic lobopodian *Hallucigenia*, which has been proposed to feed on sponges due to its limb morphology and co-occurrence with the sponge *Vauxia* (see Zhuravlev, 2001; Dunne et al., 2008). In this work, we predict a prey-predator relationship between *Hallucigenia* and *Vauxia*, as well as between *Hallucigenia* and other sponges such as *Choia*, *Takakkawia*, *Hazelia*, and *Hamptonia* (See Figure 3), therefore matching past expectations. The nature of the correlation, based on our model, suggests that *Hallucigenia* might be a generalist feeder on sponges (see Figure S8). In past literature, *Hallucigenia* has been proposed to be eaten by *Oryctocephalus* and *Parkaspis*, due to their hypostome structure, gnathobases, and limb morphology (Fortey and Owens, 1999; Dunne et al., 2008), and we recover both these interactions (Figures 3 and S8). Our model also predicts a relationship between *Hallucigenia* and *Ottoia*, with possibly the latter as a predator/scavenger on the former, as *Ottoia* is also known from fossilized gut contents to feed on epibenthic organisms such as hyoliths (*Haplophrentis*), agnostids, brachiopods (*Micromitra*), trilobites, and arthropods (*Sidneyia*) (see Vannier, 2012), and we recover most of these relationships from our analysis. We also propose that *Hallucigenia* might have been preyed upon by the anomalocaridid *Laggania* but not by other anomalocaridids; this may reflect the unique ecology of *Laggania* or it may be a spurious association due to other factors.

*Anomalocaris* and other anomalocaridids such as *Laggania* and *Hurdia* are known to be large predators with well-developed appendages, complex digestive systems, large eyes, and active swimming (Briggs et al., 1994; Daley et al., 2013; Vannier et al., 2014). Our model suggests a large repertoire of prey for them, such as smaller arthropods like *Leanchoilia*, *Sidneyia*, *Olenoides*, *Chancia*, *Emeraldella*, *Waptia*, *and Helmetia* (Figures 3 and S8). The predatory taxa could capture these smaller arthropods using their vision, spiny appendages, and swimming prowess from either nektonic or epibenthic environments. Most of these model predictions are supported by previous literature (see Dunne et al., 2008). Figure 3 outlines many such interactions between various taxa from RQ and discussing them at length would not be feasible in this work. We therefore leave it to the reader to explore the details present in this figure (and its more detailed version, Figure S8).

Results in Figure 3 only considered trophic interactions. Our correlational analyses also identified 137 possible competitive (both direct and indirect) interactions for which there is no reference set because competitive interactions are more difficult to deduce from paleo-biological data (Figure S7). Certain high correlation interactions may have been mutualisms, or based on shared environmental preference, common habitat patterns, or indirect interactions, rather than being trophic in nature (Freilich et al., 2018). We searched, unsuccessfully, for a strong habitat dependence in the fossil data (see Figure S10 and other analyses above) but still cannot rule out any of these alternative possibilities with current data. Direct fossil evidence and further paleontological knowledge is needed to verify or explore these points.

Detailed ecological analyses of correlation networks may suffer from overestimation problems (Carr et al., 2019; Freilich et al., 2018), but broader brush categorization of interactions based on abundance correlations can provide insights into the functional characteristics of fossil assemblages. Predicted interactions can be supplemented with interactions proposed by paleontological literature, based on gut contents, morphology, or other analyses, to weed out false positives. Other problems raised by earlier studies of paleoecological networks (Dunne et al., 2008; Roopnarine, 2010), such as whether correlations capture long-term prey-predator and population dynamics, were also explored here. We found that abundance correlation analyses echo results concerning long-term correlations in prey-predator models (Carr et al., 2019) and provide a strong platform for predicting species interactions without reference to prior information concerning the incidence, intensity, or character of those interactions (Figures S4 and S5). Due to these reasons, we do not go further in describing the nature and implications of the proposed interactions but rather leave it to further filtering and scrutiny by future paleo-biological studies.

Understanding ecological dynamics from fossil data has always been a major challenge, especially for older assemblages. The extraordinary fossil preservation of the Burgess Shale, including the RQ data set reported here, provides an exceptional window on possible ecological interactions during an era of major animal evolution. Past studies argued that many properties of modern ecosystem structure first emerged during the Cambrian (Bengtson, 2002), and network analyses coupled with proposed trophic interactions highlighted aspects of food web structure during this period (Dunne et al., 2008). When sufficiently strong fossil data are available, analyses of abundance correlation networks, supplemented with models to characterize biases in preservation and interpret species interactions, as outlined in our work, can reveal unknown or difficult-to-ascertain links in fossilized ecosystems and shed light on trophodynamics over evolutionary time.

### Limitations of the study

The major limitation of our approach is that biases that involve ecological or behavioral aspects of species interactions that are not expressed through a biased representation in habitat type, body type, or body size cannot be disentangled from trophic and competitive interactions. Mutualisms in particular remain unaddressed following this work. Further modeling and analysis work as well as paleontological interaction data are needed to validate or invalidate the inferences from our analysis.

### Resource availability

#### Lead contact

Further information and requests for resources should be directed to and will be fulfilled by the lead contact, Anshuman Swain (answain@terpmail.umd.edu)

#### Material availability

Not applicable.

#### Data and code availability

All relevant data needed to recreate the results are provided as supplementary material. Abundance values for all the taxa at 10 cm resolution in RQ can be found as 'abundance.csv' in Data S1; ecological habits, taxonomic affinity, body type categorization, and size metadata for all the taxa can be found as 'metadata_traits.csv' in Data S1. Relevant final data are also provided for reference—'Trophic interaction_matrix.csv' in Data S1 contains the consensus trophic interactions, and competitive_interaction_matrix' in Data S1 contains the consensus competitive/negative interactions. Simplified ABM simulation and network analysis code (along with the data) can be found at: github.com/anshuman21111/cambrian-fossil-networks.

## Methods

All methods can be found in the accompanying transparent methods supplemental file.

## Supplemental information

Document S1. Transparent methods and Figures S1–S13

Data S1. Raw data and processed information, related to Figures 1–3 Abundance values for all the taxa at 10 cm resolution in RQ can be found in ‘abundance_data.csv’; ecological habits, taxonomic affinity, body type categorization and size metadata for all the taxa can be found in ‘metadata_traits.csv’. Relevant final data are also provided for reference – ‘trophic_interaction_matrix.csv’ contains the consensus trophic interactions, and ‘competitive_interaction_matrix.csv’ contains the consensus competitive/negative interactions.

## Footnotes

Supplemental information can be found online at https://doi.org/10.1016/j.isci.2021.102271.
