# Supplementary material for: Deciphering trophic interactions in a mid-Cambrian assemblage
Source: iScience. 2021 Mar 4;24(4):102271. doi: 10.1016/j.isci.2021.102271 (PMC8010449; doi:10.1016/j.isci.2021.102271)
Supplement: Document S1. Transparent methods and Figures S1–S13 [file mmc1.pdf]

iScience, Volume *24*

Supplemental information

Deciphering trophic interactions in a mid-Cambrian assemblage

Anshuman Swain, Matthew Devereux, and William F. Fagan

Supplementary Materials

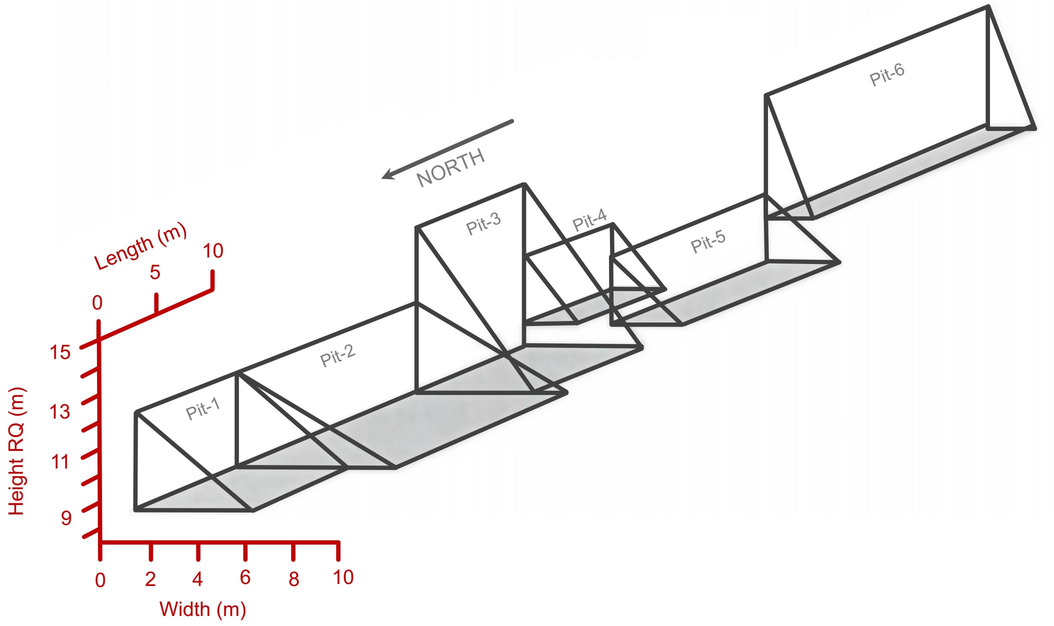

Figure S1: Raymond Quarry Excavation Schematic, related to Figure 1
Schematic of excavations in the Raymond Quarry Member. The original Raymond Quarry was located within Pit #5. The northernmost extent of Pit # l is 23 m south of the Cathedral Escarpment.

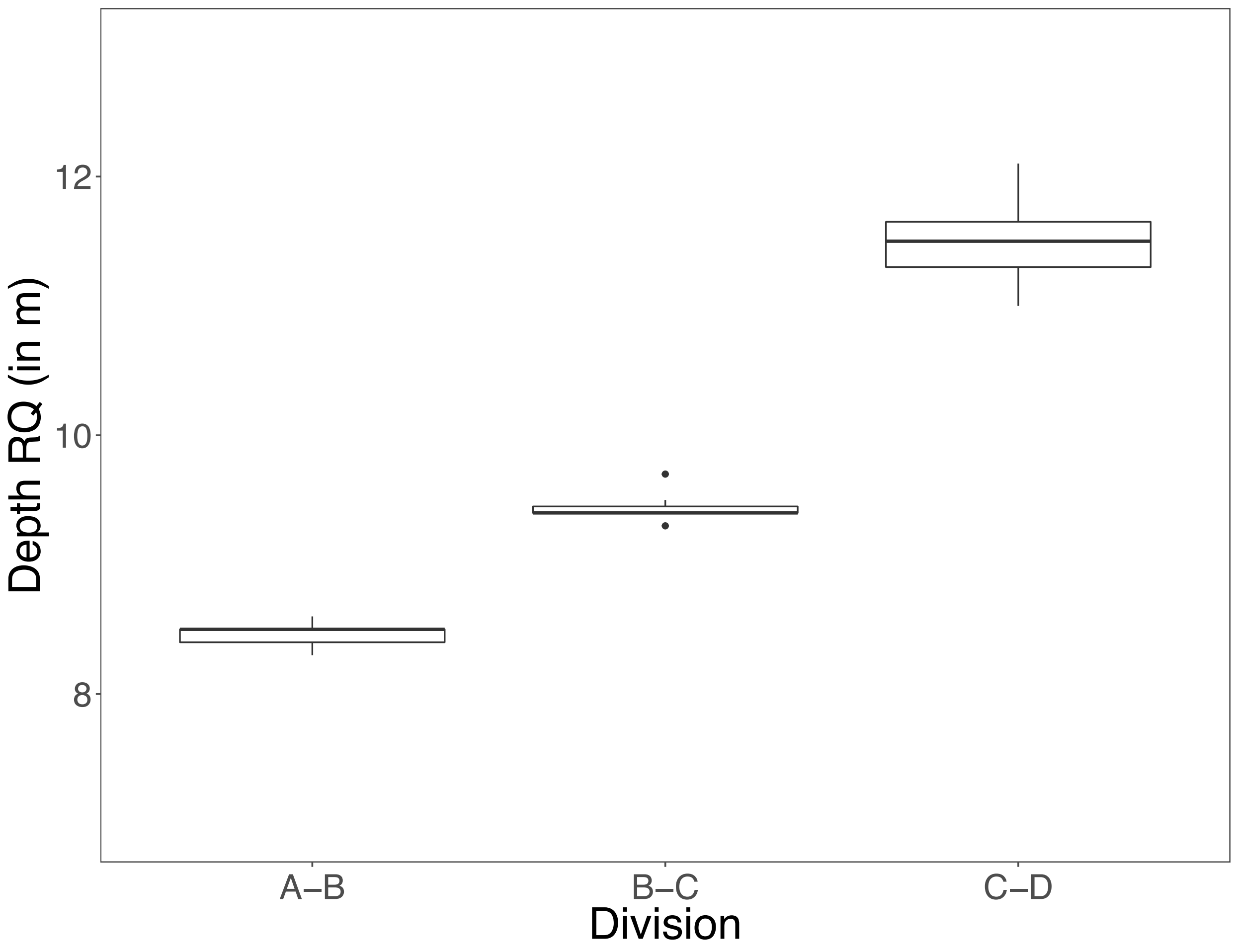

Figure S2: Biofacies detection, related to Figure 1
Biofacies detected using consensus of statistical methods ANOSIM and SHEBI for the boundaries between the Raymond Quarry sub-assemblages A-B, B-C, and C-D.

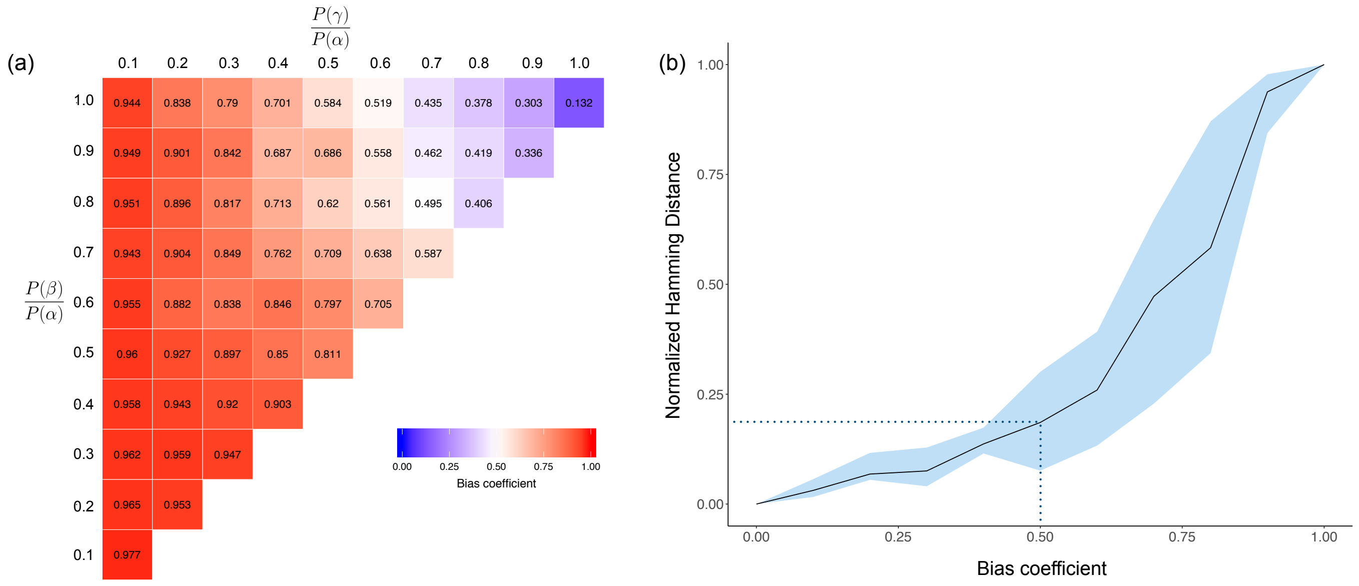

Figure S3: Preservation Bias, related to Figure 1
(a) Plot of bias coefficient as measured from the agent-based model for three categories of preservation probability: α, β and γ; (b) Differences between networks quantified using the Hamming distance (larger values imply greater deviation) as a function of the bias coefficient. The plot compares unaltered ABM networks with ABM networks whose structure was obscured by preservation biases; standard errors are calculated across alternative ABM simulations. Note the nonlinear dependence of Hamming distance on bias that increases steeply beyond bias coefficient of ~0.50.

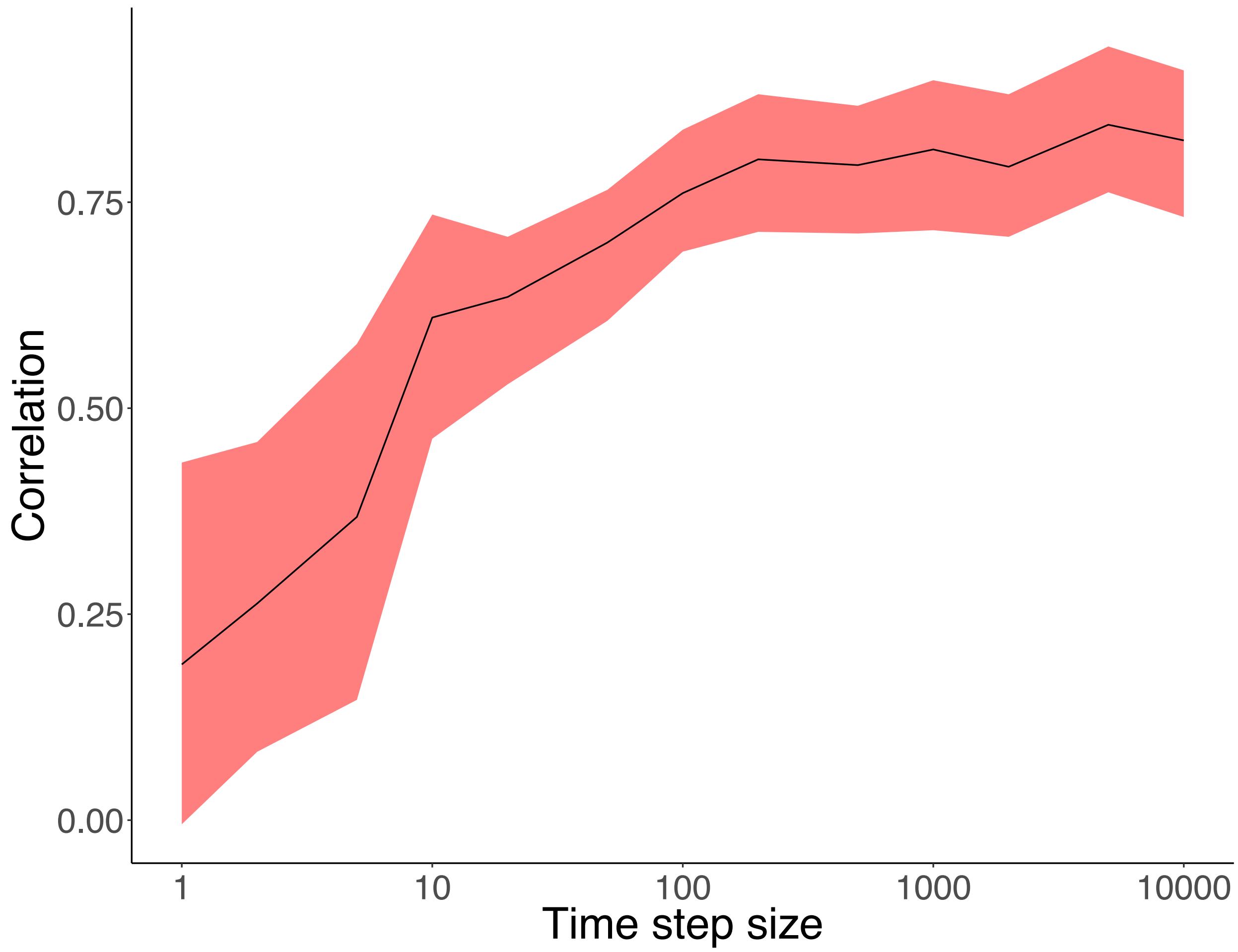

Figure S4: ABM prey-predator correlation, related to Figure 2
Correlation between a prey and its specialist predator in ABM output based on 10 sampled population sizes separated by the specified time steps. The cloud of standard error values was calculated using all the ABM runs with different initial conditions. Each ABM simulation was taken as a separate dataset and the correlation was calculated for each pair of prey and specialist predators in all the datasets. Increasing the number of sampled counts used to calculate the correlation substantially decreases the width of the error cloud.

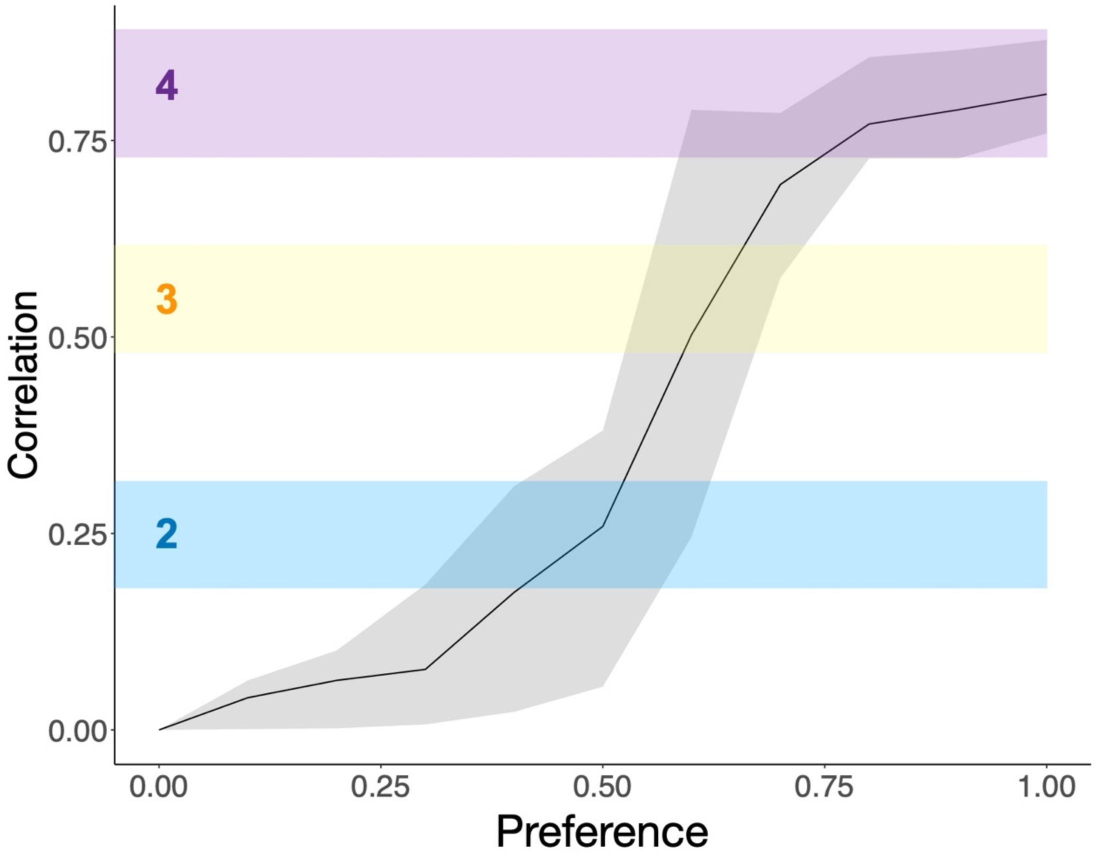

Figure S5: ABM Prey preference correlation, related to Figure 2
Correlation and preference/affinity for prey in an ABM with standard error (run for 10,000 different ABM scenarios). Mapped onto the correlation axis are the three trophic interaction categories 2, 3, and 4 from the spectral analysis.

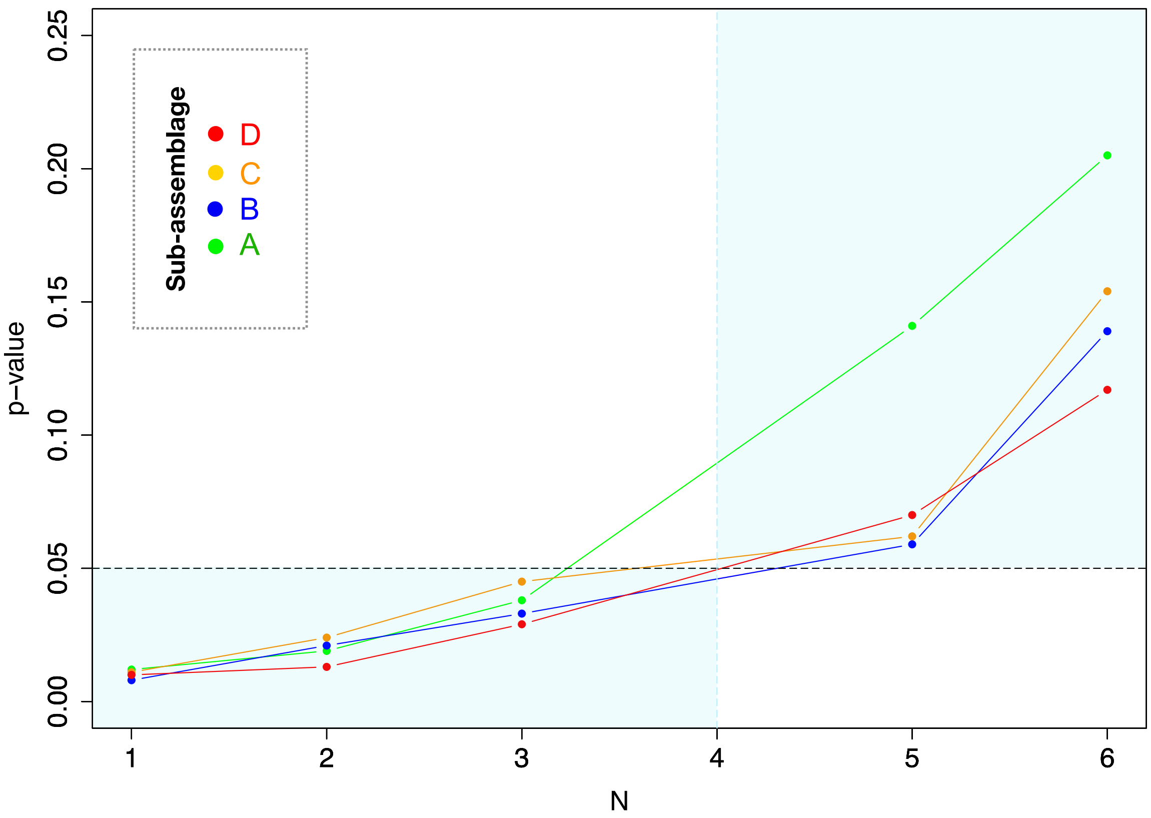

Figure S6: Optimal Clustering, related to Figure 2
p-values from chi-square test of likelihood ratio tests for nested models that differ in the number of Gaussians (N) as compared with a model with 4 Gaussians (for sub-assemblages A-D). The areas highlighted in light blue signify where the model with four Gaussians performs significantly better. All the points for all sub-assemblages lie in the region implying that a model with four basis Gaussians is the best model to pick.

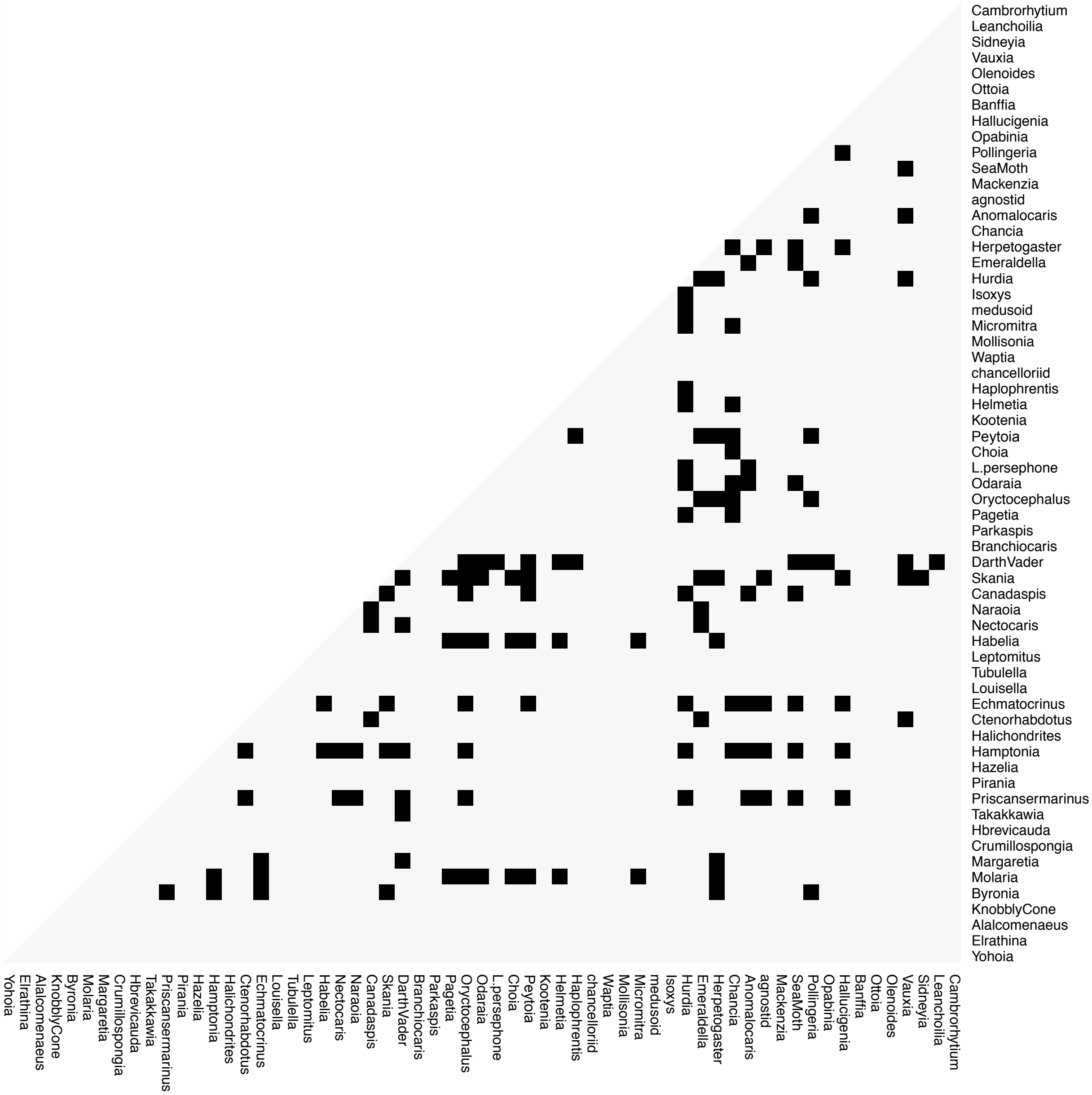

Figure S7: Competitive interactions, related to figure 2
Species interaction half-matrix representing competitive interactions among the fossil taxa proposed on the basis of abundance correlation network analyses. As it is more difficult to test competitive interactions through anatomical or other paleo-ecological evidence, we did not pursue these further, and instead focused only on trophic interactions.

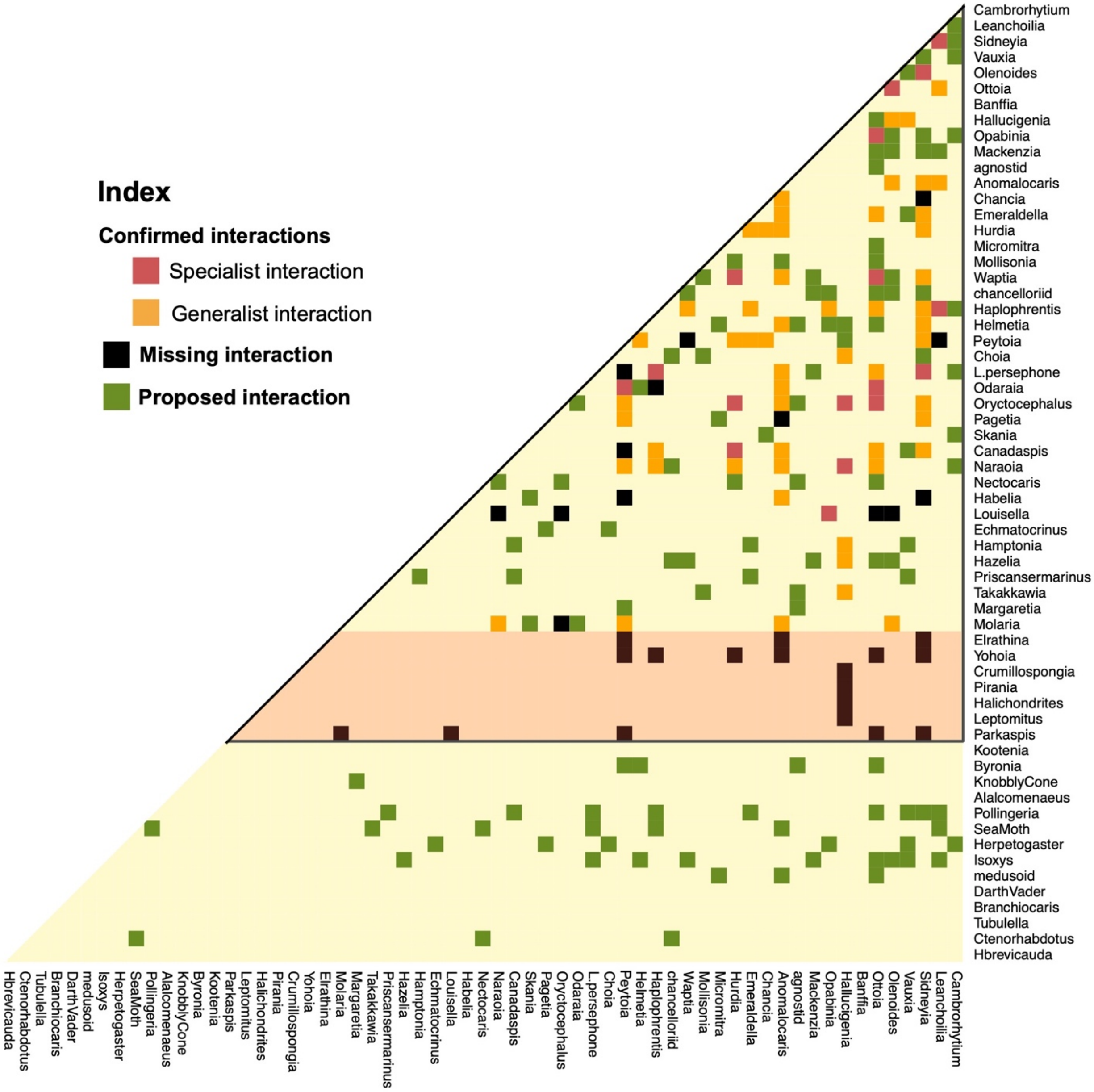

Figure S8: Detailed trophic interaction matrix, related to Figure 2 and Figure 3
Species interaction matrix showing the results from the categorization analysis, as compared with known trophic interactions from literature (Butterfield., 2000; Dunne et al., 2008; Erwin and Valentine, 2013), along with breakdown of the confirmed trophic interactions into specialist and generalist categories. Confirmed interactions were proposed in the literature and supported in the correlation analyses here. Missing interactions are reported elsewhere but did not obtain any support from our abundance correlation analyses. Proposed interactions are not currently known from the paleontological literature but are suggested by analyses here. The subset of species interactions within the black triangle are known from previous studies (Dunne et al., 2008). The species within the light orange area were numerically rare in our dataset and no statistically robust prediction could be made regarding their interactions.

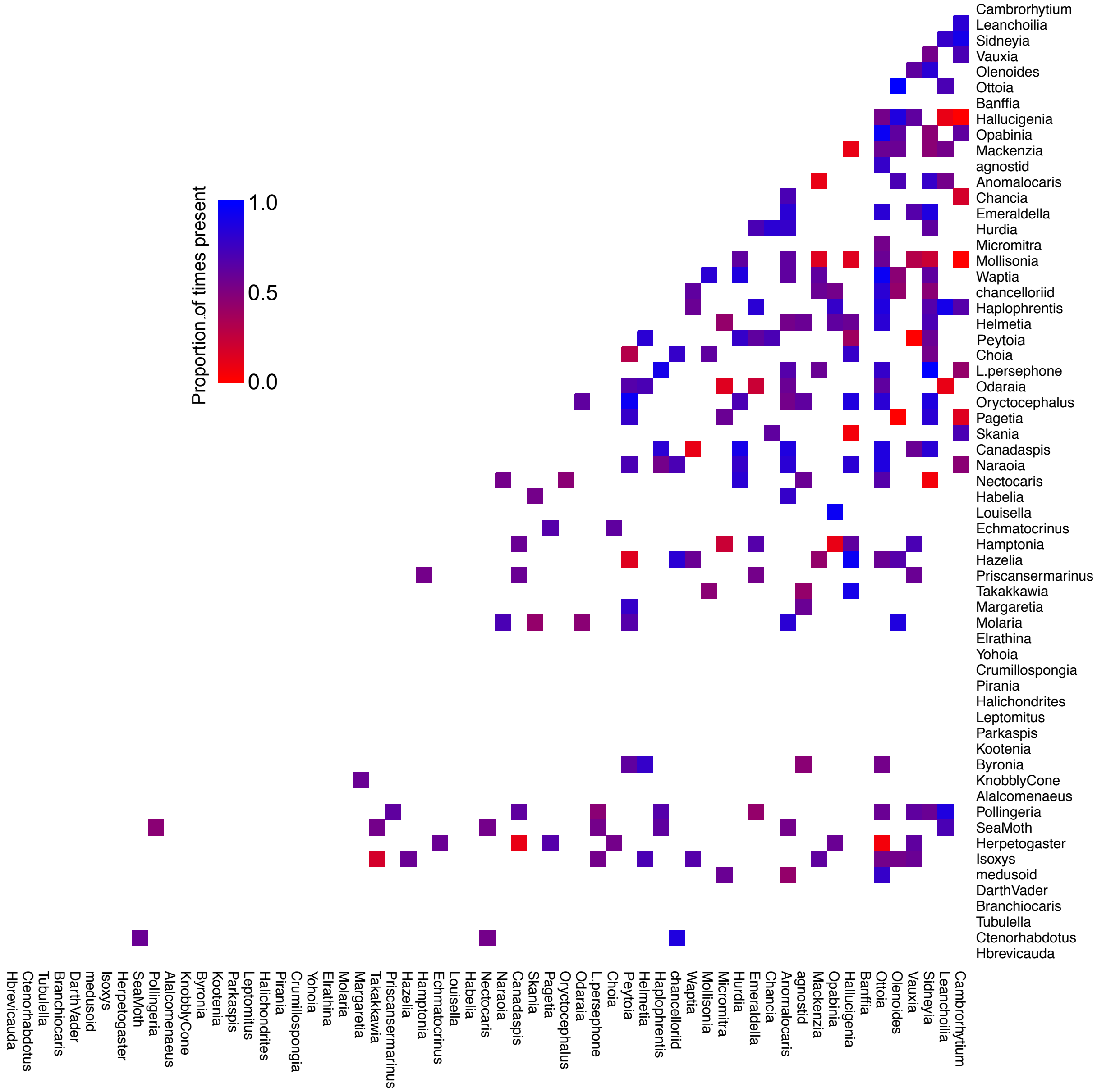

Figure S9: Interaction fidelity, related to Figure 2 and Figure 3
Heatmap showing the proportion of times high fidelity interactions were observed in the same categorization. 82.37% of the high-fidelity interactions are present consistently in the same interaction category more than 50% of the time (of co-occurrences of the pair of species) and are termed as consensus interactions. These consensus interactions are presented in the species interaction half-matrix in figure 3 and S8.

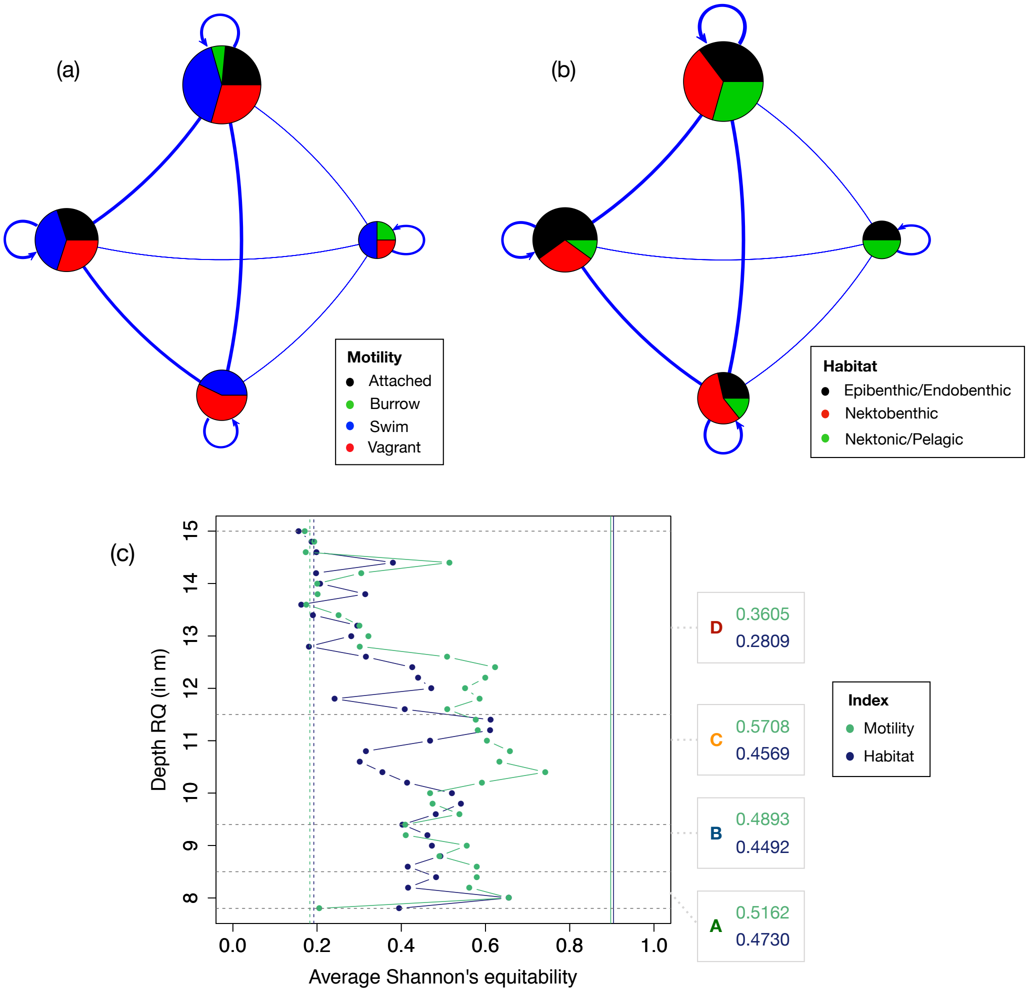

Figure S10: Stochastic block model analysis, related to Figure 1, and Figure 2
A stochastic block model of the overall network (sub-assemblages A-D) is shown here for representation purposes, which depicts four distinct blocks of taxa, estimated using integrated classification likelihood, that interact more within blocks than across blocks for motility classes (a) and habitat affinity classes (b). The lines between groups and their width denotes interaction between two blocks of taxa. Please note that the width of the self-loop here is not meaningful, other than the fact that the taxa within a block interact among themselves. The size of the block refers to the number of taxa in that block. The model has been overlaid here with (a) motility data, and (b) habitat data. Clearly, organisms belonging to particular motility or habitat classes do not fall consistently into SBM blocks. This, in turn, suggests that habitat/motility similarity does not transform into very strong associations through correlations. Please note that the number of blocks varied in different sub-assemblages and running time series analysis, as it is dependent on the structure of the network.
In (c) we use this SBM model to estimate average Shannon's equitability index (SEI) (methods) for the running timeframe analysis and for sub-assemblages (A-D). The grey horizontal dotted lines demarcate the sub-assemblages (A-D). The solid lines (separate for motility and habitat) depict the maximum theoretical value of SEI possible using our data and the dashed lines represent a theoretical SEI for 95% dominance by a single type of habitat/motility (Methods). One can see that, the SEI values depict no dependence of correlation network community structure on habitat or motility, except the start and end of the assemblage – where a weak dependence cannot be ruled out. This is in lines with the bias coefficient (methods, Figure 1(b)).

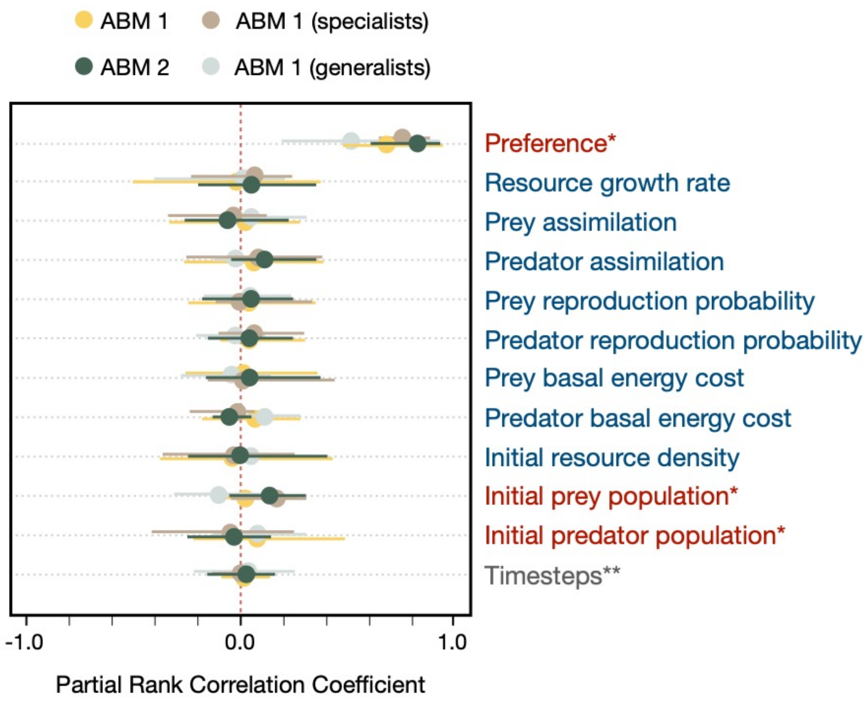

Figure S11: ABM sensitivity analysis, related to Figure 2 and Figure 3
Partial rank correlation coefficient (PRCC) of various initial conditions and parameters for the pairwise predator-prey correlations obtained in the two ABM models. Recall that ABM 1 has specialists and generalists whereas ABM 2 has a random pairwise affinity selection between prey and predators, see methods. Additional results are provided for generalists and specialists in ABM 1. The parameters/initial conditions in red are specific to a given pair of species, and the ones in blue are global parameters or initial conditions. We also examined the effect of timesteps again (see also figure S4). All parameters except resource growth rate, and basal energy costs (for prey and predator) varied between 0 and 1. Resource growth rate varied between 0 and 10, basal energy rates varied between 0.1 and 10 units. Each resource had an energy content of 1 unit. Please note that preference in case of ABM 1 was 1 for specialist interactions and 1/17 for generalist ones (as there were 17 prey species). We used a logistic transform function for PRCC categorization. The initial conditions included prey and predator populations as well as initial resource density – the last one varied between 0 and 1 (as a fraction of total area of the grid). The former two varied between 0 and 20 each. For more details, please refer to the code in the Data availability section.

We used Latin hypercube sampling (LHS) to select the parameters and initial conditions. The error bars represent 95% confidence intervals. This figure tells us that the final correlations in a wide variety of conditions were only strongly affected by the preference/categorization of interactions and were only very weakly affected by other initial conditions or parameters.

Transparent Methods

Data Collection

The primary data were collected from the Raymond Quarry in the main Burgess Shale site (Figure S1), located ~5 km north of Field, British Columbia, Canada, on a ridge connecting Mt. Field and Wapta Mountain ('Fossil Ridge') (figure S1, S2). Other exposures are known, including outcrops on Mt. Stephen that are lithologically and biostratigraphically equivalent to the Raymond Quarry Member (Fletcher and Collins 1998), such as the outcrops of equivalent strata and fossils across the Kicking Horse Valley on the shoulder of Mt. Stephen (Fletcher and Collins 1998). Consequently, the discussed fauna was not geographically isolated. Moreover, and significantly, sediment and fossil evidence support an autochthonous Raymond Quarry fauna.

Vertical bedding measurements were determined from an arbitrarily assigned RQ 10.0 m level (confirmed to be equivalent to 21.6 m above the base of C.D. Walcott's excavations in the Phyllopod Bed). This datum was traceable as a darker weathering bed across the entire exposure of the Raymond Quarry Member, except within approximately 10m of the contact with the Cathedral Escarpment, where fossils become sparse and no material was collected. Beds were subsequently measured vertically and labelled and re-labelled using a permanent marker as blocks were extracted. The first 10m mark was placed in the original Raymond quarry, carved into the rock for permanency between collecting seasons. All fossils were labelled immediately upon discovery according to the bed of occurrence, to the nearest 10cm. Specimens that occurred exactly between two measured levels were assigned to the higher level.

The majority of the physical work utilized hand-tools; no explosives were used. All the bedding planes were thoroughly and uniformly investigated. Despite the comprehensive search, adverse conditions might have rendered some fossils invisible in the field and their numbers cannot be determined exactly, but the effect would have been the same for most organisms and would have approximated their proportions recovered under more favorable conditions. For example, some *Marpolia*-like algal remains may have escaped notice, as they were usually visible only on weathered slabs (often excavated in the previous year). There is some under-representation of the macro-algal component of the community, but it was clearly always a minor contributor. As with any paleontological site, unidentifiable remains were amply seen on excavated slabs in the field. Many of these fossils were discarded unless they were deemed to possess patterns or structures that might later be identified in the laboratory, and some of these were later identified (and included among faunal counts). Shell hash lenses and other items, which appear to have been part of (transported) death assemblages, were not included in the faunal counts (for further details, please see Devereux (2001)).

Metadata for each taxon were collected using a literature survey (see references section in 'metadata_traits.csv') for the following properties: taxonomic affiliation, habitat, size, motility and preservation potential. Taxonomic affiliation was noted only when there was a majority consensus for the affiliation across sources, otherwise these were omitted from the analysis. Size data were based on the maximum size of specimens found in literature. Motility and habitat were inferred from descriptions of each taxon's anatomical characteristics. The preservation potential assignments were primarily based on literature descriptions. Hard-bodied taxa are those with biomineralized skeletons, heavily-sclerotized parts, or decay-resistant organic cuticle. Intermediate-group taxa are those with light sclerotization or unsclerotized cuticle. Soft-bodied taxa are those with soft cellular outer layers and soft internal tissues. Enigmatic metazoans (i.e. for which we have no biomineralized/sclerotized preserved parts and have no phylum consensus) were assigned to the soft-bodied group.

Identifying sub-assemblages

We used ANOSIM (Analysis of Similarity: Clark and Warwick, 1994; Bonuso et al., 2002), and SHEBI (S (species richness)-H (information) -E (evenness) analysis for Biofacies Identification: Buzas and Hayek, 1998; Handley et al., 2009) to detect consensus biofacies or sub-assemblages in the sampled region. ANOSIM is a widely used non-parametric, distance-based clustering method. Based on the rank ordering of Bray-Curtis similarities (Clarke and Warwick, 1994), ANOSIM tests for differences in community structure by mixing permutation tests with a general Monte Carlo randomization approach (Hope, 1968). SHEBI recognizes edges between samples of specimens along a (spatial or temporal) transect. It depends upon the anticipated behavior of the evenness metric, which is associated with Shannon-Weaver information, as the number of samples within a single community rises (Handley et al., 2009). Here, each sample was a 10 cm layer of shale with abundance values for each taxon. Each method was conducted on 100 bootstrap simulations of abundance for each 10 cm shale layer. The consensus values from the runs, using both methods, were pooled together, and the mean was used to define the boundaries of the sub-assemblages (named A, B, C and D from oldest to youngest). ANOSIM and SHEBI were implemented using the *vegan* (Oksanen et al., 2019) and *foramsv2.0-5* (Aluizio, 2015) packages in R, respectively.

Agent based models

Agent based models (ABMs) provide an alternative to equation-based simulations for investigating ecological scenarios in a realistic way, along with providing an easy way to incorporate spatial dependence and heterogeneity. Properly implemented, ABMs provide results that match and complement existing ecological theories and experimental evidence (DeAngelis and Grimm, 2014; Karsai et al., 2016). This led to us to choose an ABM implementation over an equation-based implementation, for representing a simple toy model of multiple prey predator interactions. Given that we are dealing with a long term (averaged) ecological abundance dataset of multiple species, there are (i) no equivalent long term complex prey-predator census dynamics of equivalent settings, and (ii) actual prey-predator relations are can only hypothesized based on paleontological evidence (see Dunne et al., 2008), we decided to use a simple ABM implementation that would have as few assumptions as possible and at the same time, also been accepted to give dynamics that have been observed in theory and experiments of prey-predator interactions (Wilensky and Rand, 1997; Tobin and Bjørnstad, 2003; Liebhold et al., 2004; Blasius et. al., 2019).

This led us to use the NetLogo language (Wilensky and Rand, 1997) and extend a Lotka-Volterra prey-predator (wolf-sheep) base model in the NetLogo library, which replicates simple ecological phenomena among prey and predators (Wilensky and Rand, 1997), to create ABMs for purposes of (a) quantifying the impacts of preservation biases, (b) calculating prey-predator correlations, and (c) categorizing interactions.

The primary simulation involved 25 species, with 17 prey and 8 predators, and was based upon an extended implementation of the base Lotka-Volterra wolf-sheep model in the NetLogo library (Wilensky and Rand, 1997). All prey fed upon a common resource, which had two parameters attached to it – rate of resource regrowth and initial density of resources (relative to the total area, which was a 500x500 grid). The resource featured an energy content, as did the prey (all prey had equal energy content for simplification). Predators were assigned either a generalist or specialist feeding style. Generalists could eat all types of prey whereas a specialist could only eat one type of prey. Energy was necessary for reproduction, and for simplicity, at each reproduction event we divided the energy between the mother and the offspring, provided that the organism had enough energy to reproduce. The rate of reproduction was controlled as a variable. One million runs of the model were conducted that involved sweeping all parameters throughout their ranges using the Latin hypercube sampling method. Each model ran until it reached a stable state (only resource was left, all predators died, or all of the organisms died) or it reached 50,000 time points, whichever occurred first. Data were transferred to R for further processing using the

package *RNetLogo* (Thiele, 2017). Each of these models were used as a dataset for (a) testing bias, (b) calculating prey-predator correlations, and (c) categorizing interactions.

The second model was the same as the first/primary model except that each predator species was randomly assigned a different preference for each prey (between 0 and 1). This preference was the probability of a predator consuming a given prey when encountered. One million runs of the model were conducted that involved sweeping all parameters throughout their ranges. Each model ran until it reached a stable state (only resource was left, all predators died, or all of the organisms died) or it reached 50,000 time points, whichever occurred first.

The main reason for performing the ABM simulations was to obtain time series data pertaining to populations of various species, such that their relative fluctuations could tell us more about their trophic and competitive interactions. In other words, system stability or convergence in any form was not necessary for our model, but rather to reduce the computational time, we looked for states where it was no longer meaningful to continue the simulations to 50,000 iterations. The set of simulations that terminated early included cases where only resources persisted or where all species went extinct. From an initial pilot study, we saw a range of extremely complex patterns in populations, and observed slightly different outputs even with the same initial conditions. These results were due to the stochasticity inherent in our spatial ABM models, and we accommodated this variability by increasing the number of runs of the simulations.

To understand the effect of different parameters on the resultant correlations, as well as the effect of different lengths of time on the correlations, we performed partial rank correlation coefficient (PRCC) analysis in Figure S11. We ran 1 million simulations with different initial conditions and parameter settings based on a Latin hypercube sampling paradigm and observed the dependence of the final prey-predator correlation. We did not observe any strong dependence of pairwise correlations on initial conditions or parameter values except for the parameter controlling the preference/categorization (specialist or generalist) of the prey-predator pairs. A list of all the parameters and initial conditions are provided in Figure S11.

Data from all ABM simulations were transferred to R for further processing using the package *RNetLogo* (Thiele, 2017). Each of these models was then used as a dataset for categorizing interactions.

Constructing networks

Fossil count data from two adjacent 10 cm layers were combined in each sub-assemblage to increase species coverage for network construction. For each 20cm unit of each sub-assemblage (A through D, excluding A' and D' as in Figure 1 on the basis of preservation bias), we iteratively sampled fossils using bootstrap process for 1000 iterations. Using the data for each iteration, we calculated mean correlations between distinct 20 cm blocks for each sub-assemblage across all the bootstrap replicates. Given that some of these interactions can be spurious, we applied partial correlation corrections to the correlation matrices of each sub-assemblage (for details on partial correlation corrections in networks – see Epskamp and Fried, 2018). This ensured that when a third variable is associated with two measurements, after eliminating the effect of that variable, through partial correlation correction, we are able to provide robust estimate for association as compared to the existing raw correlation, i.e., this step corrects for spurious correlations arising from indirect effects (such as correlation between A and B because both are correlated with C). Next, we performed a Fisher Z-transform of the partial correlation matrices and calculated the probability of observing the estimated Z-scores by chance (based on a normally distributed null distribution). Finally, we used the Benjamini-Hochberg correction of p-values to eliminate those

interactions whose corrected correlation p-values exceeded 0.01. This yielded the final set of high-fidelity interactions for each sub-assemblage.

We also created networks on a running time-frame basis where we started at the beginning of the assemblage and repeated the process of network construction as described above for all possible 1.2 m sub-sections created by shifting the analysis frame in 20cm increments.

Pair-wise correlations were calculated for each dataset of the agent-based model simulations and across differently sized time steps as slices for calculating correlations (data were aggregated for each slice) (see Figure S2). We took 100 time-steps as the benchmark for each time slice for constructing networks for all purposes as the correlation saturated at a high enough value and the effects of phase difference and noise were reduced at this time scale of simulations.

Testing Preservation Bias using Exponential Random Graph Models

Exponential Random Graph Models (ERGMs: Holland and Leinhardt, 1981) are a preferred tool for evaluating how individual variables shape network structures. ERGMs have been used in the past to look at missing data and bias (Robins et al., 2004). We used the *ergm* (Handcock et al., 2019) package in R.

Using the trophic ABM datasets, we assigned each species in each simulated dataset to one of three preservation categories (α, β, or γ) to create a new 'partially preserved' dataset whose abundance values were adjusted downward by fixed preservation probabilities where

$$1 \geq P(\alpha) \geq P(\beta) \geq P(\gamma) \geq 0$$

These three categories can be thought of differential preservation categories: for example, in case of body type: hard bodied (preserves well like α), intermediate bodied (preserves decently but less than hard body, like β) and soft bodied (preserves poorly as compared to other body types; can be denoted by γ).

We repeated this procedure 100 times each for all 1 million simulated datasets and then constructed corresponding networks for each of the original and partially preserved datasets. For each of these constructed networks (both altered/partially preserved and original), we calculated the dependence of the network structure on the preservation category (α, β or γ) using ERGMs. We calculated the p-value resulting from the ERGM model for both the altered (partially preserved) networks and the original (intact) networks, as well as their mutual Hamming distance.

The bias coefficient (B) is measured in terms of these ERGM p-values of the original and partially preserved networks ($p_{original}$ and $p_{partially\ preserved}$ respectively) as

$$B = \frac{p_{original} - p_{partially\ preserved}}{p_{original}}$$

B > 0.5 on this scale corresponds to a change of ~0.2 of Hamming distance (Figure S3). Note that the ABM simulations were used to validate this statistical method before we applied it to the fossil data.

Moreover, this framework can detect biases in network structure based on categorizations, even when the relative preservation potentials among the categories is unknown, but only the categorizations are known. This is a useful property because, although we can assign relative ease of preservation in categorizations of say, body type – assigning the same for habitat and body size might be more complex (see supplementary file 'metadata_traits.csv').

Using this framework, we calculated ERGM p-values for each of the networks from the running time-frame analysis, as well as the four sub-assemblages (A-D) of the fossil data. Because we do not have the structure for the unaltered network of the fossil data (i.e. the actual abundance correlation networks from when the burial happened), we assume $p_{original} = 1.0$ for these analyses, in order to calculate the bias coefficient. This assumption gives an upper bound on the bias coefficient for our data, as the minimum possible ERGM p-value would be represented by $p_{original} = 1.0$ scenario (i.e. no dependence of network structure on any factor), but the actual p-value would usually be lower than this assumed value. As, all reported bias coefficients for the fossil data is based on this assumption, they represent the 'worst-case' values.

We performed three sets of analysis in this regard: body type, body size, and habitat affiliation. In each set, there were three categories, which were pre-determined (see supplementary file 'metadata_traits.csv' for details), according to available paleontological evidence. The body type categorizations were based on preservation or fossilization potential, as described in 'Data collection' sub-section of Methods – namely, hard bodied, soft bodied and intermediate, based on literature descriptions. Body size categorizations were <15 cm, 15 - 30 cm, > 30 cm maximum size. Habitat types were categorized into endobenthic/epibenthic, nektobenthic, nektonic/pelagic, based on literature survey (see references section in 'metadata_traits.csv').

Categorization of interactions and comparison with ABM

We subjected the distributions of abundance correlations to a maximum likelihood analysis to identify appropriate Gaussian basis functions. The mean, and standard deviation for a pre-defined number of Gaussian basis functions were determined using a general simplex-based optimization algorithm (Nelder and Mead, 1965). We compared results assuming 1 through 6 possible Gaussian basis functions and identified the optimal number of such functions for the correlation data using the likelihood-ratio test. All the scripts were implemented in R.

Once the number and nature of the Gaussians were estimated (for sub-assemblages A-D and for the running time-frame analysis), we used pairwise Kolmogorov-Smirnov (KS) tests between basis Gaussians to obtain a pairwise similarity matrix. The number of clusters of Gaussian basis functions was determined using spectral analysis based on the pairwise similarity matrix obtained from KS analysis and the gap statistic (Tibshirani et al., 2000). An advantage of using the gap statistic is that it does not pre-assume the number of required clusters (Tibshirani et al., 2000). All scripts were implemented in R using the package *cluster*. Four zones of clustering were determined using this method and are termed categories of interactions. Each category is shown in Figure 2 (a) using the range of all associated Gaussian means.

Next, from the ABM datasets, we identified the nature (i.e., generalist-prey, specialist-prey, competition, apparent competition) of all interactions whose partial correlations fell within ranges of the Gaussian mean clusters and plotted their relative occurrences in Figure 2c.

Consensus interactions were calculated from the running time-frame analysis and were defined as the high-fidelity interactions (or statistically corrected correlations) that stayed in the same interaction category for more than 50% of the time it occurred for a given pair of taxa. These results have been plotted in figures 3, S8 and S9.

Stochastic Block Models (SBMs) and Equitability analysis

The stochastic block model (SBM) is a tool, used to detect community structure in a network, where communities can be defined as multi-node subcomponents (or, blocks) of the network in which edges are more common within than between communities (Karrer and Newman, 2011).

We applied the framework of SBMs to the networks constructed from fossil data to understand the associations among species. In particular, we sought to understand whether the correlations on which those networks were built represented shared motility or habitat variables rather than species interactions. At each network level (which were calculated at a sub-assemblage level A-D and also at a fine time scale level), we used integrated classification likelihood (ICL) to calculate the number of clusters/blocks. On each block/cluster, we annotated the taxa in those blocks using the metadata on habitat and motility (separately; see supplementary file 'metadata_traits.csv' for details) and used the *mixer* package (Latouche et al., 2012) to find the distribution of annotated categories across blocks at a given network level. In order to numerically represent it, we calculated Shannon's equitability index (SEI), which is the normalized Shannon's diversity coefficient, based on the categories of habitat/motility for each block/cluster – and to estimate a network level (for a sub-assemblage/fine time scale analysis) average – we found the weighted average (on basis on number of taxa in each block/cluster) of SEI over all the blocks/clusters in the given network. We then plotted this average network SEI value at the fine scale analysis level in Figure S10(c).

SEI points at the dominance of a specific type of say, habitat/motility, on the taxa involved in interactions within a calculated cluster. If a given cluster is highly dominated by a single type of habitat/motility, SEI would be very low and would be 0 if it is only type. As SEI is normalized diversity index, the highest possible value of 1 occurs when all the types are equally probable. This is not the case with our fossil data – and hence, we calculated the maximum empirical value possible with the data (Figure S10(c)) for both habitat and motility separately. In addition, to make sense of how biased the correlational values are, we calculated the SEIs, for both habitat and motility, where a dominant type is equal to 95% of a given cluster and others are equally distributed in the remaining fraction (Figure S10(c)).

Supplemental References

Aluizio R. (2015). forams: Foraminifera and Community Ecology Analyses. R package version 2.0-5. https://CRAN.R-project.org/package=forams

Bonuso, N., Newton, C. R., Brower, J. C., & Ivany, L. C. (2002). Does coordinated stasis yield taxonomic and ecologic stability?: Middle Devonian Hamilton Group of central New York. Geology, 30(12), 1055-1058.

DeAngelis, D. L., & Grimm, V. (2014). Individual-based models in ecology after four decades. F1000prime reports, 6.

Epskamp, S., & Fried, E. I. (2018). A tutorial on regularized partial correlation networks. Psychological methods, 23(4), 617.

Fletcher, T. P., & Collins, D. H. (1998). The middle Cambrian Burgess Shale and its relationship to the Stephen Formation in the southern Canadian Rocky Mountains. Canadian Journal of Earth Sciences, 35(4), 413-436.

Handcock, M.S., Hunter, D.R., Butts, C.T., Goodreau, S.M., Krivitsky, P.N. and Morris, M. (2017). ergm: Fit, simulate and diagnose exponential-family models for networks. The Statnet Project (http://www. statnet. org). R package version, 3(0).

Handley, J. C., Sheets, H. D., & Mitchell, C. E. (2009). Probability models for stasis and change in paleocommunity structure. Palaios, 24(10), 638-649.

Holland, P.W. and Leinhardt, S. (1981). An exponential family of probability distributions for directed graphs. Journal of the american Statistical association, 76(373), 33-50.

Hope, A.C. (1968). A simplified Monte Carlo significance test procedure. Journal of the Royal Statistical Society: Series B (Methodological), 30(3), 582-598.

Karsai, I., Montano, E., & Schmickl, T. (2016). Bottom-up ecology: an agent-based model on the interactions between competition and predation. Letters in Biomathematics, 3(1), 161-180.

Latouche, P., Birmelé, E. and Ambroise, C. (2012), Variational Bayesian inference and complexity control for stochastic block models. Statistical Modelling, SAGE Publications, 12, 1, 93-115.

Nelder, J. A. and Mead, R. (1965). A simplex algorithm for function minimization. Computer Journal, 7, 308–313.

Oksanen, J., Blanchet, F. G., Kindt, R., Legendre, P., Minchin, P. R., O'hara, R. B., ... & Wagner, H. (2013). Community ecology package. R package version, 2(0).

Thiele J.C. (2017). RNetLogo: Provides an Interface to the Agent-Based Modelling Platform 'NetLogo'. R package v.1.0-4. https://cran.r-project.org/package=RNetLogo

Wilensky, U., & Rand, W. (2007). Making models match: Replicating an agent-based model. Journal of Artificial Societies and Social Simulation, 10(4), 2.
